# Quantitative Analysis of Molecular Mobility in Amorphous Lactose Above *T_g_*: A Novel Insight from Molecular Dynamic Simulation to *Strength* Parameter

**DOI:** 10.3390/foods14060928

**Published:** 2025-03-08

**Authors:** Fanghui Fan, Huan Liu, Yier Xu, Tian Mou

**Affiliations:** 1 Department of Food Science and Engineering, College of Chemistry and Environmental Engineering, Shenzhen University, Shenzhen 518060, China; fanghui.fan@szu.edu.cn (F.F.); 2310222056@email.szu.edu.cn (H.L.); 2021140012@email.szu.edu.cn (Y.X.); 2 School of Biomedical Engineering, Shenzhen University Medical School, Shenzhen University, Shenzhen 518060, China

**Keywords:** molecular mobility, molecular dynamic simulation, root mean square displacement, amorphous lactose, *Strength* parameter, water

## Abstract

Measuring molecular mobility (*M_m_*) in solid food is challenging due to the rigid and heterogeneous nature of these matrices. The thermodynamic parameter *Strength* (*S*) fails to account for molecular displacement distances. This study emphasizes the role of molecular dynamic (*MD*) simulation in quantifying *M_m_* on amorphous lactose at mimic water activities (*a_w_*) at temperatures above the glass transition temperature (*T_g_*), incorporating the *S*. The results show that coordinating root mean square displacement (*RMSD*) effectively quantifies *M_m_* across different *a_w_* and temperature conditions. Both increased *a_w_* and higher temperatures facilitate *M_m_* by expanding free volume and reducing energy barriers for molecular rearrangement, as indicated by the mobility coefficient calculations. This study also emphasizes the importance of system size in interpreting *M_m_*, as larger systems exhibit emergent behaviors that smaller systems cannot capture. The calculated *MD* relaxation time for 10,000-molecule lactose/water cells at a specific *S* value was successfully translated to a real timescale of 1.8 × 10^6^ s, consistent with experimental data (1.2 × 10^6^ s). Moreover, water can shift from a plasticizing role to a more stabilizing one, slowing molecular motion and leading to equilibrium clustering. These findings have important implications for understanding the behavior of amorphous lactose in food and pharmaceutical formulations.

## 1. Introduction

The molecular mobility (*M_m_*) of amorphous sugars is a critical factor in determining physicochemical stabilities and processability of solid foods [1,2]. As the surrounding temperature surpasses the glass transition (*T_g_*) of amorphous sugars, pronounced changes occur, including increased entropy and heat capacity, alongside decreased rigidity and viscosity, which collectively alter the *M_m_* of the sugar-containing foods [3]. It should be noted that the glass transition represents a kinetic relaxation process tied to the temperature-sensitive relaxation behavior of amorphous materials governed by *M_m_* [4]. Recent findings suggest that carbohydrates’ molecular structures affect this relaxation, likely driven by molecular motions within matrix heterogeneities [5]. In amorphous lactose, for instance, *α*- and *β*-relaxation processes are linked to motions of side groups and carbon backbones due to the availability of free volume above *T_g_* [6]. However, the non-equilibrium nature of amorphous sugars complicates the quantification of *M_m_*, necessitating an integrated approach that combines both thermodynamic and kinetic perspectives.

Considering the rigid and heterogeneous nature of amorphous sugars, building on proper measurements of *M_m_* for amorphous sugars is challenging due to the complex motions. Such complexity encompasses molecular displacement from solvent migration or mechanical strain, Brownian motion, and the movements of atomic groups, facilitating reactant displacement that can trigger crystallization or degradative reactions in solid foods [7]. Taking an asymmetric lactose molecule as an example, which has 129 vibrational modes, its *M_m_* involves three translational and rotational modes, and 3*N* − 6 vibrational modes, where *N* is the number of atoms in the molecule [8]. In addition, the dynamic complexity arises from vibrational, rotational, and translational mobility modes inherent to amorphous sugars, compounded by the heterogeneous nature of food matrices, which often include crystalline, amorphous, and semi-crystalline phases, as well as diverse interactions with water. Each component uniquely contributes to the overall *M_m_*, complicating its measurement, where *M_m_* is significantly restricted and less observable than in a fully amorphous state [9]. These complexities underscore the necessity for advanced integrative approaches to accurately quantify the *M_m_* within the diverse and intricate environments of amorphous sugars.

Water, as a small-molecule plasticizer, exerts a profound influence on the *M_m_* of amorphous sugars, as even minor variations in local water content can significantly accelerate or decelerate sugar molecular movements [8]. These changes have far-reaching implications for degradation reactions, shelf life, and the sensory attributes of amorphous sugar-containing foods [10]. To quantify the impact of water on the *M_m_* of amorphous sugars, the *Strength* (*S*) parameter has been introduced as an innovative thermodynamic descriptor [11]. Derived from Williams–Landel–Ferry (*WLF*) modeling, the *S* parameter defines the critical temperature difference above *T_g_* at which abrupt changes in material properties occur, providing a valuable tool for characterizing *M_m_* under typical processing and storage conditions [4]. This framework complements the *Deborah* number, which can translate relaxation times (*τ*) into real timescales, offering insights into the effects of composition on relaxation phenomena above the measured *T_g_* [12]. However, the *S* parameter only captures the energetic requirements for shifts in the average motion distribution of amorphous sugars. It does not account for molecular displacement distances or diffusion coefficients, underscoring the need for more comprehensive molecular-scale investigations to deepen our understanding of mobility in amorphous sugars.

Recent analytical techniques, such as thermodynamic and spectroscopic methods like nuclear magnetic resonance, proton-induced X-ray emission or gamma-ray emission, infrared spectroscopy, differential scanning colorimeter, *Raman* spectroscopy, terahertz time-domain spectroscopy, etc., are essential for probing *M_m_* in amorphous sugars but often face limitations in capturing molecular-scale displacement [13]. Complementing experimental methods, employing molecular dynamic (*MD*) simulations and quantum mechanical calculations provides valuable insights into molecular interactions and processing effects, which are difficult to observe experimentally [14]. With the ever-growing computer power and the development of theoretical models, the *MD* suggests a new way to track molecular trajectories in the quantum mechanical models. For example, *MD* allows for high-precision modeling of molecular behavior at atomic levels, enabling researchers to visualize phase transitions, such as glass transition and crystallization, and predict how factors like water, temperature, and composition affect the *M_m_* of the system [15]. Furthermore, it can mimic spectroscopic data, facilitating comparisons between computational predictions and experimental results. Therefore, the combination of *MD* and experiment enhances understanding of *M_m_* to accommodate the heterogeneous nature of matrices, supporting efforts to optimize solid food processing, extend shelf life, and improve food quality. Few studies have focused on the application of the *MD* approach in exploring the *M_m_* and often ignore the heterogeneous nature of amorphous sugars and their interactions with water.

Previous studies pointed out that the sensitivity of the *S* parameter in amorphous lactose (β-D-galactopyranosyl (1–4)-D-glucopyranose) to surrounding water activities (*a_w_*) often exhibited a high correlation with the quality deteriorations of lactose-containing foods, such as crystallization [16], caking [17], volatiles loss [18], etc. In this paper, the simulant lactose/water cells were built with varying molecular numbers (from 100 to 10,000), and corresponding molecular trajectories were tracked at mimic *a_w_* (0.11 to 0.44) and temperature differences above *T_g_*. We aim to introduce an *MD* approach for quantifying *M_m_* in amorphous lactose and investigate water’s role in modulating sugar displacement at atomic scales. In addition, the thermodynamic *S* parameter of amorphous lactose and the correlation between *S* and molecular displacements were examined, providing a theoretical framework to bridge experimental and computational expressions of *M_m_* in amorphous lactose. This work offers a promising solution for measuring *M_m_* in amorphous sugars. Also, it highlights the complex interactions between food components, emphasizing the dependence of relaxation-related mobility behaviors on external factors such as *a_w_* and temperature, as well as the intrinsic physical states of the solid foods.

## 2. Materials and Methods

### 2.1. MD Simulation

The amorphous lactose cells were constructed, and atomic simulations were performed using Materials Studio 2020 (version 20.1.0.2728; BIOVIA, San Diego, CA, USA), with varying molecular numbers to mimic water activities ranging from 0.11 to 0.44 *a_w_* and temperature differences (≥*T_g_* with 10 K intervals up to *T_g_* + 30 K at studied *a_w_*). In this study, the *MD* simulation was complemented by a supercomputer in the National Supercomputing Center (Shuguang 6000 supercomputer equipped with 1024 CPU and internal memory of 800 GB). The brief simulation procedure is as follows:

*Structure Building* Lactose and water molecules were built using the “*Sketch*” menu and idealized at coarse-grained scale by bead–spring models.

*Cells Construction* Constructing the cubic model using an “*Amorphous Cell*” module. Four types of simulant cells were constructed to mimic studied *a_w_* including molecular numbers from 100 to 10,000; the detailed molecular ratios can be found in Table 1.

*Structure Optimization* Use the “*Geometry Optimization*” in the “*Forcite*” module with the COMPASS III force field as well as the constant number of molecules, volume, and temperature (*NVT*) dynamics to perform structure optimization of simulant cells. Considering to the objectives of the study, which aims to analyze the effects of temperature variations on the displacement of lactose/water system, the *NVT* is chosen for investigations focused on properties at a specific volume or when temperature regulation is paramount.

*Production Run* After the optimization stage, production runs were carried out at studied water activities and temperatures. The time step was defined as 1 *fs* for all the dynamic runs, and the molecular dynamic simulation was performed for 100,000 steps up to 100 ps, with molecular trajectory data recorded every 1000 steps. While longer simulations would ideally provide more detailed insights into diffusion behavior [19], our preliminary tests demonstrate that simulations up to 1000 ps exhibit trends consistent with those observed at 100 ps (Figure S1). Anderson thermostat and Berendsen barostat were used to regulate temperature and pressure, respectively. The velocity Verlet algorithm is used to solve the equations of motion, and the “*Ewald*” method is applied to manage long-range interactions using a buffer width of 2 Å.

**Figure 1:**
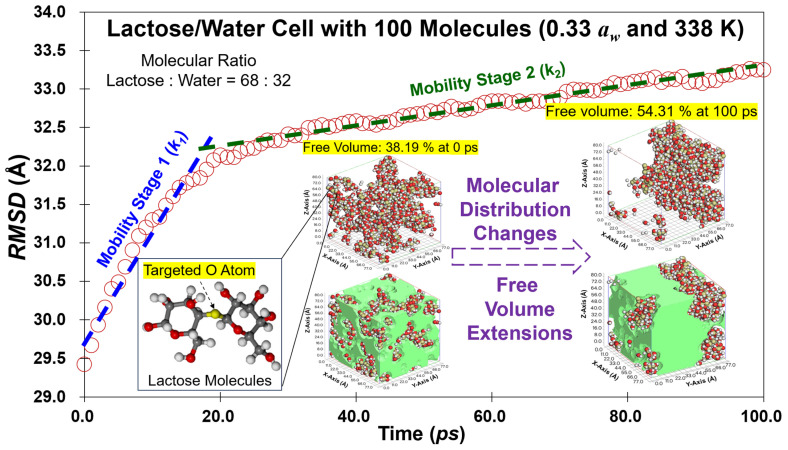
Schematic depiction of the *RMSD* for simulant lactose/water cell with 100 molecules in total at mimic 338 K and 0.33 *a_w_*. The changes in molecular distribution and visible free volume (green space) were presented based on the *MD* calculation at different times.

*Coordinate Extraction* A *Perl* script (Version 5.10.0; Perl Foundation, Holland, MI, USA) sorts the trajectory of lactose and water molecules in the simulant cells after running up to 100 ps. To track the molecules, the oxygen atom was chosen as the tracking target at the geometric centers of lactose and water molecules (Figure 1). These oxygen atoms represent the coordinates of the lactose and water molecules and are extracted at each simulation step.

*Free Volume Calculation* The total volume and free volume data of optimized simulant cells for the studied *a_w_* values and temperatures are calculated using *Multiwfn* software (Version 3.8; Beijing, China) [20,21]. Since the size, density, and measurement parameters of simulant lactose/water cells are fixed in this paper, the free volume is a certain value.

### 2.2. M_m_ Measurement

As noted above, the *MD* simulation is a computational technique grounded in classical mechanics, where molecules are treated as particles characterized by their coordinates, masses, charges, and chemical bonding. The initial positions and velocities of these molecules are assigned based on a Boltzmann random distribution [22]. Utilizing a COMPASS III force field potential, this study computed the forces and interaction energies between particles, allowing for the determination of each particle’s velocity and acceleration through the numerical solution of Newton’s equations of motion. This process updates the coordinates of the particles iteratively, and the cycle is repeated for a predetermined number of simulation steps. The output consists of molecular coordinates and velocities over time, which collectively form the phase-space trajectories. These trajectories are then analyzed using statistical physics and thermodynamic principles to extract relevant physical properties of the system. Root mean square displacement (*RMSD*) serves as a statistical metric to quantify molecular motion over time (Equation (1)). Within the context of *MD* simulations, *RMSD* is widely used to characterize the extent of molecular displacement in Cartesian space. For amorphous solids, higher *RMSD* values are indicative of greater molecular diffusion and enhanced *M_m_*, whereas lower *RMSD* values signify restricted motion or slower dynamics. *RMSD* thus provides a robust measure of *M_m_* and its dependence on system properties, offering valuable insights into dynamic behavior at the molecular level. In this study, the *RMSD* was used to analyze *M_m_* of amorphous lactose/water matrix in *MD*-built cells after equilibrium up to 100 ps.

(1) $$RMSD=\sqrt{\frac{1}{N}\sum_{i=1}^{N}\delta_{it}^{2}}$$

where *N* is the number of molecules in the system, and $\delta_{it}^{2}$ is the distance between molecule *i* and the mean position of the *N* equivalent atoms at time *t*. Since this study focuses on how water influences lactose movement, separating displacement calculations clarifies these interactions and enhances the ability to draw meaningful conclusions about molecular behavior. Therefore, the mean position of *N* water and *M* lactose molecules is defined as follows in Equation (2):

(2) $$\mu_{t}=\frac{1}{N+M}\sum_{i=1}^{N}\sum_{j=1}^{M}{(w_{it}+l}_{jt})$$

The distance between water or lactose and the mean position is defined in Equations (3) and (4).

(3) $$\delta_{it}^{Water}\;=\;{(w}_{itx}-\mu_{tx},w_{ity}-\mu_{ty}{,w}_{itz}-\mu_{tz})$$

(4) $$\delta_{jt}^{Lactose}\;=\;{(l}_{jtx}-\mu_{tx},l_{jty}-\mu_{ty}{,l}_{jtz}-\mu_{tz})/r$$

where *r* is the ratio of molecular size between lactose and water (1.18/0.13 = 9.08) calculated in the *MD* cell-building procedure. Then, the total *RMSD* is shown in Equation (5).

(5) $${RMSD}_{t}\;=\;\sqrt{\frac{1}{N+M}\sum_{i,j=1}^{N,M}{\left(\delta_{it}^{water}\right)}^{2}+{\left(\delta_{jt}^{lactose}\right)}^{2}}$$

### 2.3. Mobility Coefficient Calculation

The mobility coefficients are calculated from the well-known Einstein relationship by the least squares linear fitting of the linear portion of mean square displacement (*MSD*) curves (Equation (6)).

(6) $$D=\frac{1}{6N}\underset{t\to \infty }{\lim}\frac{d}{d_{t}}MSD_{t}$$

where *D* is the mobility coefficient, and *N* is the total number of molecules in simulant cells. It should be noted that the *RMSD* represents the square root of the *MSD*, providing a direct measure of average displacement in linear units. In contrast, the *MSD* gives the average squared displacement, which is less intuitive for interpreting physical distances due to its squared unit scale. This study focuses on calculating the water and lactose mobility, which reflects the average displacement of entire simulant cells containing multiple molecules. By selecting *RMSD*, therefore, we aim to facilitate straightforward comparisons of length scales between water and lactose molecules. Figure 1 shows the *RMSD* of simulant lactose/water cells against time calculated from *MD* simulation. The graphs were fitted using linear regression, *y* = *kx* + *b*, and the slope of the regression line, *k*, can be obtained (Figure 1). In this paper, therefore, Equation (6) can be simplified to *D* = *k*/6.

### 2.4. Activation Energy Calculation

Having the mobility coefficient data over a range of temperatures allows us to calculate the activation energy (*E_a_*) of mobility for molecules. The *E_a_* can be calculated using the Arrhenius equation (Equation (7)):

(7) $$D=D_{0}\exp\left(-\frac{E_{a}}{RT}\right)$$

where *D* is the mobility coefficient (cm^2^∙s^−1^) at temperature *T* (K), *D*_0_ is a pre-exponential factor, *R* (8.314 J∙mol^−1^∙K^−1^) is the universal gas constant, and *E_a_* is the activation energy (J∙mol^−1^).

### 2.5. Strength Parameter Measurement

The measurement of the *S* parameter in amorphous lactose at the studied *a_w_* range (0.11 to 0.44) was sourced from our previous studies [4]. The *S* value of the system is determined by Equations (8) and (9), where *C*_1_ and *C*_2_ refer to the material-special *WLF* constants. The *Deborah* number refers to a decrease in the number of logarithmic decades for flow, e.g., to result in stickiness, can be defined as the critical parameter (*d_s_*), and a corresponding (*T* − *Tg*) is given as the strength of the solids, *S* parameter. It should be noted that the *S* parameter of carbohydrate–polymeric food systems could be calculated at *d_s_* = 4 [23].

(8) $$\mathrm{Log}\left(\frac{\tau }{\tau_{g}}\right)=\frac{-C_{1}(T-T_{g})}{C_{2}+(T-T_{g})}$$

(9) $$S=\frac{d_{s}C_{2}}{-C_{1}-d_{s}}$$

### 2.6. Statistical Analysis

The *RMSD* of triplicate measurements was analyzed by the *R* program (version 4.4.1; R Core Team, Vienna, Austria) and Microsoft Excel (2019, Microsoft, Inc., Redmon, WA, USA). The average values with a standard deviation of triplicate measurements were calculated. Additionally, the error bars and significance analysis were implemented in the confidence interval of 95 % to represent the variability of data.

## 3. Results

### 3.1. Mobility Trajectories

**Figure 2:**
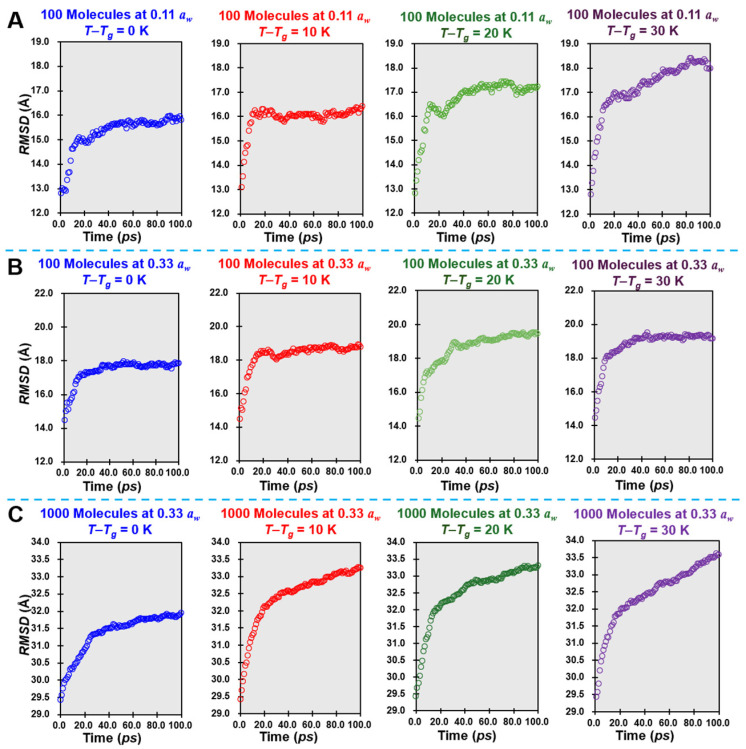
The changes in the *RMSD* of simulant lactose/water cells at studied temperature differences ((**A**,**B**) for 100 molecules at mimic 0.11 and 0.33 *a_w_*; (**C**) for 1000 molecules at mimic 0.33 *a_w_*).

Following the MD simulations (Figure 2), the *RMSD* versus time plots for simulant lactose/water cells were generated to assess molecular trajectories across different mimic water activities and corresponding temperature differences (*T − T_g_* from 0 to 30 K). Figure 2A,B show a system of 100 molecules at *a_w_* = 0.11 and 0.33, while Figure 2C illustrates a larger system of 1000 molecules at *a_w_* = 0.33 in temperature differences studied. As observed in the *RMSD* trajectories, an increase in *a_w_* from 0.11 to 0.33 leads to a notable rise in *RMSD*, indicating enhanced *M_m_* [24]. This trend aligns with the established understanding that higher *a_w_* facilitates *M_m_* due to the increased availability of free water molecules, which act as plasticizers and reduce system rigidity [25]. Additionally, as the temperature rises above *T_g_*, the mobility of the molecules increases, as reflected in the steeper slope of the *RMSD* curve at higher temperatures. This is indicative of the thermally activated nature of molecular motion, where higher temperatures provide the energy needed to overcome barriers to molecular rearrangement, thereby increasing the rate of displacement. The *RMSD* curves show a rapid linear increase up to 20 ps, followed by a plateau until 100 ps, suggesting that a stable displacement is achieved within the simulant cells. These configurations offer valuable insights into how water and molecular numbers modulate *M_m_*, with implications for understanding the stability and reactivity of amorphous lactose.

In the low mimic *a_w_* (Figure 2A), the limited availability of water molecules creates a tightly constrained molecular environment, reducing mobility and then showing antiplasticization effects. Here, water molecules are primarily bound to lactose, forming hydration shells that stabilize the matrix but restrict molecular freedom through strong hydrogen bonding interactions. This results in a viscous environment, characteristic of highly concentrated amorphous systems, and is crucial for stabilizing dehydrated products like powders and freeze-dried formulations [8,26]. In contrast, Figure 2B shows a significant increase in molecular mobility at *a_w_* = 0.33, where water acts as a plasticizer, reducing intermolecular forces and increasing the dynamic range of motion for both lactose and water molecules. This enhanced mobility could accelerate undesirable processes, such as lactose crystallization or enzymatic hydrolysis, in food and pharmaceutical systems [27]. The transition from 100 molecules to 1000 molecules introduces additional complexities, such as cooperative interactions and the effects of localized hydration, which may further alter dynamic behavior (Figure 2C). Larger cells may exhibit collective phenomena, such as cooperative relaxation or phase separation, which influence *M_m_*. These findings emphasize the importance of considering cell size and molecular composition when interpreting *MD* simulations and caution against directly extrapolating small-scale studies to larger, real-world systems.

### 3.2. Mobility Coefficients and Activation Energy

Table 1 presents the mobility coefficient (*D_m_*) and activation energy (*E_a_*) for simulant lactose/water cells constructed by 100 molecules, at various mimic water activities and temperature differences, based on *MD* calculations. The data offer insights into the relationship between *a_w_*, temperature above *T_g_*, and *M_m_* in amorphous lactose. The mobility coefficients (*D_m_*_1_ and *D_m_*_2_) show a clear dependence on both *a_w_* and temperature differences. At *a_w_* = 0.11, for example, the mobility coefficients are relatively low, with *D_m_*_1_ ranging from 0.0405 Å/ps at 338 K (*T_g_*) to 0.0562 Å/ps at 368 K (*T − Tg* = 30 K). These values suggest limited *M_m_*, consistent with the constrained molecular movement observed at low *a_w_*, where strong interactions between water and lactose molecules hinder diffusion. As *a_w_* increases (0.22 and 0.33), mobility coefficients rise, indicating enhanced *M_m_*. For instance, at *a_w_* = 0.33, *D_m_*_1_ increases from 0.0298 Å/ps at 313 K (*T_g_*) to 0.0335 Å/ps at 323 K (*T − Tg* = 10 K), reaching a maximum of 0.0361 Å/ps at 316 K (*T − Tg* = 30 K) for *a_w_* = 0.44. This increase is consistent with the role of water as a plasticizer, which reduces intermolecular forces and facilitates greater molecular movement. The activation energies (*E_a_*_1_ and *E_a_*_2_) provide further insights into the temperature dependence of *M_m_*. Higher activation energies are generally associated with systems where molecular rearrangements require more energy to overcome barriers to mobility. For example, at *a_w_* = 0.11, *E_a_*_1_ ranges from 10.99 kJ/mol to 12.81 kJ/mol, indicating that higher energy is needed for mobility at low *a_w_*. In contrast, at *a_w_* = 0.33, *E_a_*_1_ is lower, ranging from 6.94 kJ/mol to 9.45 kJ/mol, reflecting reduced energy requirements for molecular movement as water content increases. This trend underscores the plasticizing effect of water, which weakens hydrogen bonding between water and lactose molecules, lowering the energy barriers for molecular rearrangements.

These findings align with the trends observed in the *RMSD* trajectories, where *M_m_* increases with *a_w_* and temperature differences above *T_g_*. Higher water activities and temperatures correlate with increased mobility coefficients and lower activation energies, further highlighting the role of water as a plasticizer. Additionally, the temperature dependence of mobility coefficients and activation energies underscores the thermally activated nature of *M_m_*, where higher temperatures facilitate molecular rearrangements by providing the necessary energy to overcome intermolecular forces. Moreover, the transition from 100 molecules to larger systems (e.g., 1000 molecules) introduces additional complexities, such as cooperative relaxation and phase separation, which may further impact *M_m_*. These scaling effects emphasize the need to consider both *a_w_* and system size when studying the molecular dynamics of amorphous lactose. In conclusion, the data presented in Table 1 confirm the critical role of water as a plasticizer in modulating *M_m_*. The observed increase in mobility coefficients and decrease in activation energies with higher water activity and temperature differences above *T_g_* have significant implications for the stability and reactivity of lactose-based systems, particularly in food and pharmaceutical applications where water and temperature are key factors influencing product quality and shelf life.

### 3.3. Free Volume in Cells

**Figure 3:**
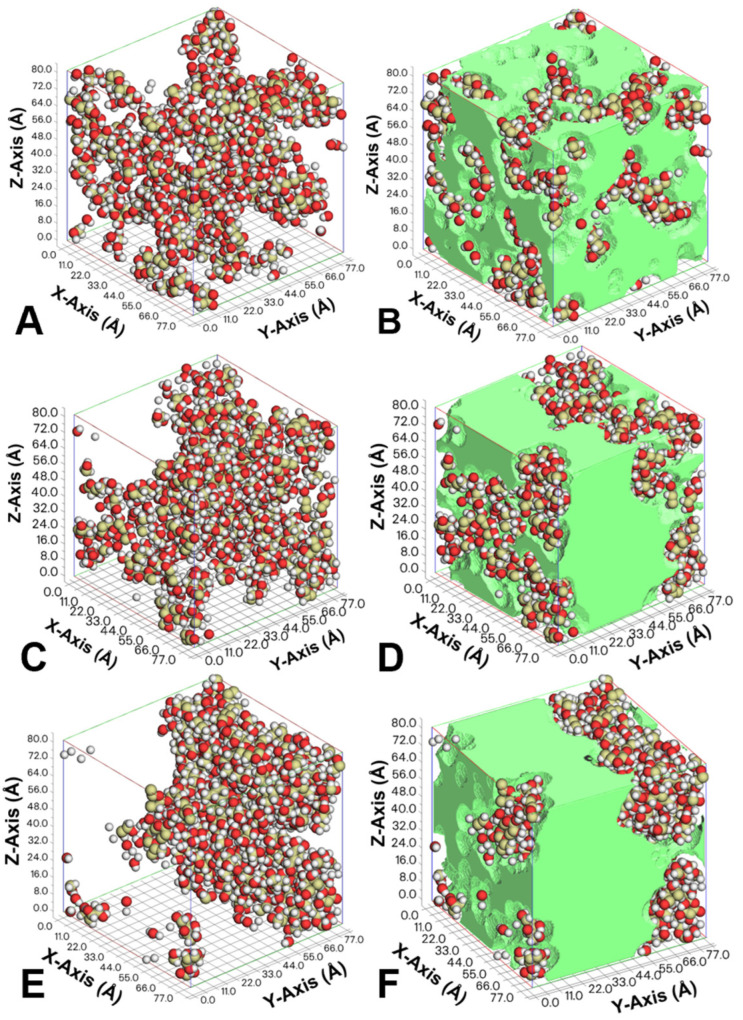
The movements of lactose and water molecules in simulant cells with 100 molecules at different simulation times (0 ps: (**A**,**B**), 20 ps: (**C**,**D**), 100 ps: (**E**,**F**)) were calculated at 0.11 *a_w_* and 338 K. The green part is marked as the free volume in simulant cells.

Previous studies reported that the influence of temperature on molecular mobility could be explained in terms of free volume theory [28]. In this study, a common probe method is used to calculate the free volume of simulant lactose/water cells, where the Connolly surface is determined by rolling the probe molecule over the van der Waals surface. The free volume is then defined as the volume on the side of the Connolly surface without atoms. Figure 3 illustrates the movement of lactose and water molecules in simulant lactose/water cells, constructed with 100 molecules, over different times and temperatures. The *3D* visualizations in Figure 3 show distinct changes in *M_m_* as a function of temperature and *a_w_*. At lower temperatures and water activities (e.g., *a_w_* = 0.11), the molecular movement appears restricted, with lactose and water molecules occupying localized regions. As both temperature and *a_w_* increase, as seen in Figure 3C–F, molecular movement becomes more pronounced, with water molecules spreading more uniformly throughout the system. This enhanced mobility is consistent with the trends observed in the *RMSD* data (Figure 2), where higher temperatures and water activities correlate with increased molecular diffusion. Table 2 and Table S1 further support this observation, providing calculated free volume data for simulant lactose/water cells at various water activities and temperatures. As the *a_w_* increases, the free volume also increases, reflecting the plasticizing effect of water. For instance, at *a_w_* = 0.11, the free volume at 0 ps (initial time) is 3220.939 Å^3^, while at *a_w_* = 0.33, it rises to 33,265.822 Å^3^, suggesting that higher *a_w_* reduces the rigidity of the lactose matrix, thus enhancing *M_m_* (Table 2). The percentage of free volume, which indicates the proportion of the system’s volume available for molecular movement, also increases with higher water activities, supporting the observed rise in *M_m_*, as indicated by mobility coefficients and activation energy.

**Figure 4:**
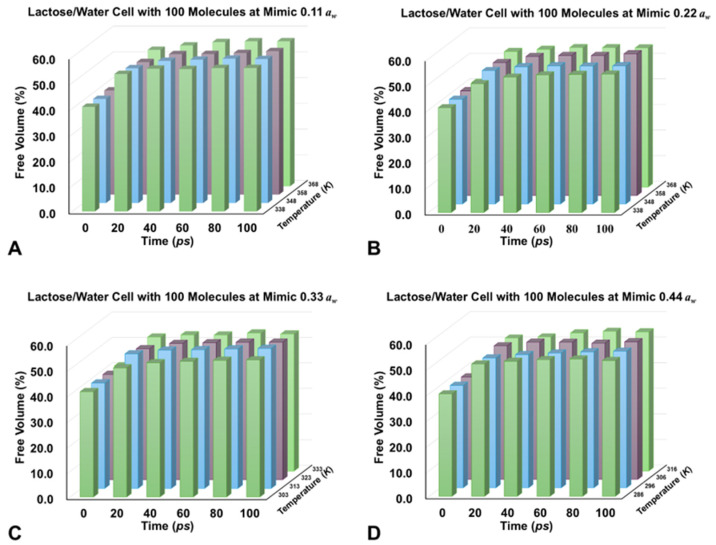
The changes of free volume from 0.11 to 0.44 *a_w_* (**A**–**D**) during 100 ps molecular dynamics simulation of simulated lactose/water cells constructed from 100 molecules at studied temperature differences (*T* − *T_g_* from 0 to 30 K).

Figure 4 presents the changes in free volume over time for simulant lactose/water cells constructed with 100 molecules at various simulated water activities (0.11 to 0.44). It should be noted that the rapid fluctuations in free volume observed over short timescales are primarily attributed to two main factors: the fixed box size constraint and insufficient pre-equilibration. In this study, the quick changes in the free volume of pre-equilibration lactose/water simulant cells during the simulation stem from maintaining a constant particle density throughout the process (Table 2). This constraint implies that any variations in molecular configurations or dynamics can lead to noticeable changes in free volume. Even after completing pre-equilibration steps, the system may still exhibit significant fluctuations during the initial stages of the main simulation run. Figure 4A–D illustrate the evolution of free volume as a function of simulation time and temperature for different *a_w_* levels. At lower water activities (0.11 and 0.22), the free volume increases slowly over time, reflecting the limited mobility of the system, as seen in Figure 4A,B. This behavior is consistent with the earlier observations where, at low *a_w_*, lactose molecules are tightly bound to water, forming a stable matrix with limited mobility (Figure 2A). At these low hydration levels, the molecular environment remains rigid, with water molecules primarily serving to stabilize the system through hydrogen bonding, which limits molecular movement and reduces free volume. As the *a_w_* increases Figure 4C,D, there is a more pronounced increase in free volume, especially at 0.33 and 0.44 *a_w_*, reflecting enhanced *M_m_*. This trend aligns with the findings in Table 1, where higher water activities were associated with higher mobility coefficients and lower activation energies, indicating increased molecular movement. The larger free volume suggests that more water molecules are available to reduce the rigidity of the lactose matrix, allowing for greater *M_m_* and facilitating dynamic molecular rearrangements. This supports the role of water as a plasticizer, which weakens intermolecular forces and promotes mobility within the system, as described in the *RMSD* trajectories (Figure 2). The trends observed in this study align with the observations in the *RMSD* data and activation energy analysis, emphasizing the thermally activated nature of molecular motion and the antiplasticizing effect of water.

The temperature dependence of *M_m_* is also evident in both the free volume data and the molecular movement visualizations. As temperature increases, the free volume expands, resulting in more available space for *M_m_* [29]. This behavior is particularly evident at higher temperatures, such as 316 K, where the free volume increases significantly, leading to enhanced *M_m_*. This is consistent with the thermally activated nature of *M_m_*, as higher temperatures provide the energy necessary to overcome barriers to molecular rearrangement, thereby facilitating faster diffusion and greater mobility, as reflected in the mobility coefficients and the visualized molecular movement in Figure 3. The transition from smaller cells (100 molecules) to larger systems introduces additional complexities. While the mobility coefficients and free volume provide a clear picture of *M_m_* in smaller systems, larger systems may exhibit collective behaviors, such as cooperative relaxation or phase separation, which can further influence *M_m_*. These results emphasize the importance of considering both system size and molecular composition in interpreting the dynamics of amorphous lactose/water systems, as larger systems may exhibit emergent behaviors not captured in smaller systems. Collectively, the data from the *MD* simulations, free volume calculations, and *RMSD* analyses underscore the significant role of *a_w_* and temperature difference above *T_g_* in modulating *M_m_* in lactose/water systems.

### 3.4. S Parameter and M_m_

**Figure 5:**
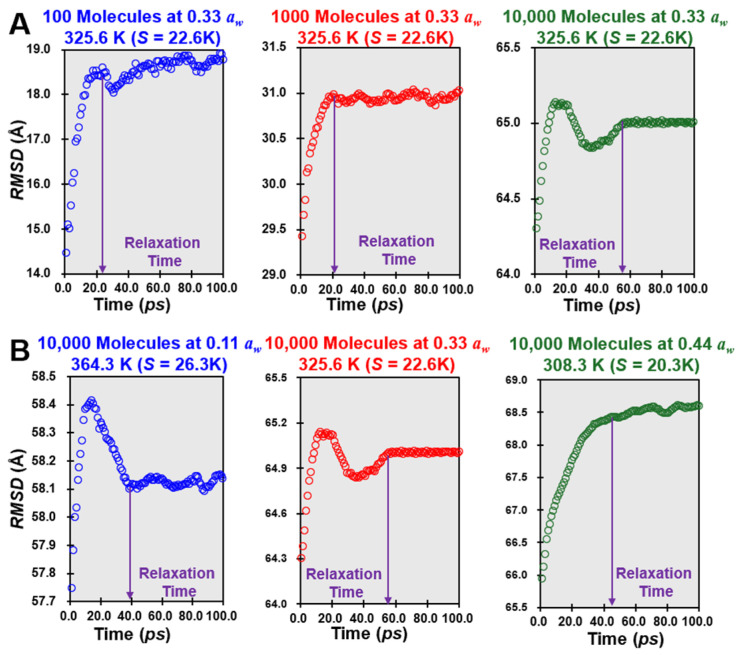
The changes in the molecular mobility of different lactose/water matrices ((**A**) refers to the size of cells from 100 to 10,000 molecules, simulating the *S* value at 0.33 *a_w_*; (**B**) for 10,000 molecules in simulant cells, simulating the *S* value from 0.11 to 0.44 *a_w_*).

Figure 5 demonstrates the *M_m_* changes in simulant lactose/water cells as a function of *a_w_* and system size, analyzed through the *S* value, a parameter translating measured relaxation times to real timescales. Figure 5A represents a system from 100 to 10,000 molecules with an *S* value corresponding to *a_w_* = 0.33, while Figure 5B encompasses a larger system of 10,000 molecules, with the *S* value varying across a range of mimic *a_w_* values (0.11 to 0.44). In Figure 5A, the *S* value encapsulates *M_m_* at a fixed *a_w_* (0.33) within both small and big molecular systems. This configuration highlights the direct impact of system size on relaxation dynamics. At 0.33 *a_w_* level, water molecules begin acting as plasticizers, weakening intermolecular forces and increasing the mobility of lactose molecules. Compared to 10,000 molecules, the system’s relatively small size (100 molecules) ensures limited molecular complexity, emphasizing individual interactions rather than collective dynamics. These results demonstrate that at *a_w_* = 0.33, relaxation times are substantially increased compared to cells with 100 molecules, indicating a transition from a rigid to a more dynamic molecular matrix. Figure 5B expands on the observations in Figure 5A by examining a larger system of 10,000 molecules across a broader range of *a_w_* (0.11 to 0.44). The difference in system size between Figure 5 also raises important considerations about scaling and heterogeneity. While smaller systems (Figure 5A) provide insights into localized molecular interactions, larger systems (Figure 5B) introduce emergent behaviors, such as clustering and cooperative relaxation, that are relevant to real-world applications. This distinction underscores the importance of considering both scale and hydration when applying these findings to practical formulations.

The *S* value here captures the nonlinear relationship between *a_w_* and *M_m_*. At *a_w_* = 0.11, the restricted availability of water molecules limits their plasticizing effect, resulting in longer relaxation times and reduced *M_m_*. However, as *a_w_* increases, the *S* value decreases, signifying enhanced relaxation dynamics due to the increased mobility of both water and lactose molecules. This behavior underscores the pivotal role of water in breaking hydrogen bonds and facilitating molecular interactions. Beyond *a_w_* = 0.33, further increases in *a_w_* (up to 0.44) amplify these effects, leading to a pronounced acceleration in molecular dynamics. This pattern demonstrates the critical threshold effect of *a_w_* on relaxation times, with the *RMSD* values providing a quantitative measure of the extent of *M_m_* across different hydration states. It should be noted that The *S* parameter provides a quantitative framework for correlating relaxation times with real timescales, making it a practical tool for predicting behavior in amorphous sugars at *a_w_* below 0.44. Moreover, the critical parameter (*d_s_*) in S parameter calculation (Equation (9)) refers to a decrease in a number of logarithmic decades for flow in the amorphous lactose, which may eventually lead to crystallization. Previous studies have shown that the real timescale for full crystallization of 1 g of amorphous lactose occurs over approximately 14 days (1.2 × 10^6^ s) at *a_w_* ranging from 0.11 to 0.44, at around 20 °C above *T_g_*, where α-lactose monohydrate predominates in the system [4]. In this study, for *a_w_* = 0.33 and *S* = 22.6 K, the calculated relaxation times for simulant lactose/water cells with 10,000 molecules are approximately 2.0 × 10^−11^ s. When considering Avogadro’s constant (6.0 × 10^23^), these relaxation times can be translated to a real timescale of 1.8 × 10^6^ s, which is consistent with experimental observations [30]. This demonstrates that the S parameter provides a robust framework for translating experimental relaxation times into real-world timescales, offering a more intuitive understanding of molecular mobility in amorphous sugars.

### 3.5. Lactose Mobility Pathways

**Figure 6:**
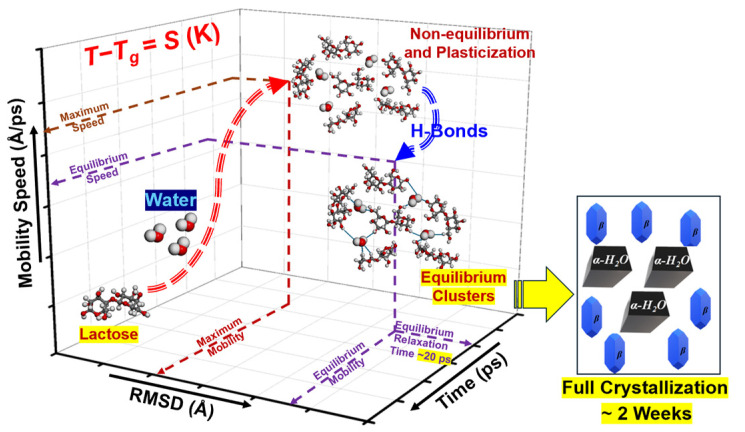
Schematic diagram for the *M_m_* pathways of the lactose molecules under hydration conditions based on the results of *MD* simulations.

Figure 6 provides a schematic illustration of the mobility dynamics of lactose molecules in the presence of water, emphasizing the role of water in modulating molecular displacement. The plot presents a *3D* representation of mobility speed versus *RMSD* and time, with water playing a critical role in altering the mobility behavior of lactose molecules. To be specific, Figure 6 depicts the dynamic process in which water first induces a non-equilibrium plasticization effect, enhancing the mobility of lactose molecules. This initial phase is marked by increased displacement, as water molecules reduce the intermolecular forces within the lactose matrix, allowing for greater molecular movement [8]. As more water molecules interact with lactose after approximately 20 ps, they begin to form hydrogen bonds (H-bonds), leading to the formation of equilibrium clusters. These clusters represent a transition from the initial plasticization phase to a more stable state, where lactose molecules are more tightly bound by water, reducing their mobility [31]. The formation of these equilibrium clusters decreases the displacement and mobility of the amorphous lactose, ultimately reaching an equilibrium state characterized by relative lower mobility and a longer relaxation time. This transition highlights the dual role of water in lactose systems. Initially, water acts as a plasticizer, promoting molecular mobility by reducing the rigidity of the system. However, as the system evolves, water molecules form H-bonds with lactose, creating stable clusters that restrict molecular displacement. This process parallels the physical behavior of amorphous lactose, where water not only enhances initial molecular movement but also induces a reduction in mobility once the system reaches equilibrium. Ultimately, the observed decrease in mobility over time due to H-bond formation aligns with the concept that water molecules can shift from a plasticizing role to a more stabilizing one, slowing molecular motion and leading to equilibrium clustering. This dual effect of water is critical for understanding the molecular behavior of amorphous lactose in various applications, particularly in dehydrated food and pharmaceutical formulations. For example, restricting mobility through low hydration can prevent crystallization and degradation, enhancing the stability of powdered and lyophilized formulations. Conversely, leveraging the dynamic state at higher hydration levels can optimize processes like dissolution and controlled drug release.

## 4. Conclusions

This study shows that coordinating root mean square displacement effectively quantifies *M_m_* across different *a_w_* and temperature conditions. Both increased *a_w_* and higher temperatures facilitate *M_m_* by expanding free volume and reducing energy barriers for molecular rearrangement, as indicated by the mobility coefficient calculations. This study also emphasizes the importance of system size in interpreting *M_m_*, as larger systems exhibit emergent behaviors that smaller systems cannot capture. The calculated *MD* relaxation time for 10,000-molecule lactose/water cells at a specific *S* value was successfully translated to a real timescale, consistent with experimental data. Moreover, water can shift from a plasticizing role to a more stabilizing one, slowing molecular motion and leading to equilibrium clustering. These insights have significant implications for the stability, texture, and reactivity of lactose-based products, offering valuable guidance for optimizing formulations in food and pharmaceutical applications. However, the current study still shows some limitations, such as the shortness of simulation time and huge calculation time consumption during the *MD* measurement. Future studies would build on this conceptual framework by integrating experimental data and optimizing the computational simulations to quantitatively map the transition points and dynamic states in the amorphous sugar/protein matrix at a longer simulation time range.

## Figures and Tables

**Table 1:** The molar ratios of lactose (L) and water (W) molecules, mobility coefficient (*D_m_*), and activation energy (*E_a_*) for simulant lactose/water cells constructed by 100 molecules in total at mimic water activities (*a_w_*) and temperature differences (*T* − *T_g_*) based on *MD* calculation. The *T_g_* sourcing from the experimental calorimetric onset data [4].

Simulated *a_w_*	Ratio (L:W)	*T* − *T_g_* (K)	*k*_1_	*R*_1_^2^	*k*_2_	*D_m_*_1_ (Å/ps)	*D_m_*_2_ (Å/ps)	*E_a_*_1_ (kJ/mol)	*E_a_*_2_ (kJ/mol)
0.11	68:32	0	−0.2432	0.9589	0.0118	0.0405	0.0002	10.9878	12.8061
		10	−0.2931	0.9618	0.0098	0.0489	0.0016		
		20	−0.3219	0.9527	0.0061	0.0537	0.0010		
		30	−0.3371	0.9691	0.0011	0.0562	0.0002		
0.22	57:43	0	−0.1687	0.9846	0.0027	0.0298	0.0005	8.3431	10.1664
		10	−0.1090	0.8231	0.0055	0.0317	0.0009		
		20	−0.4255	0.9885	0.0013	0.0350	0.0005		
		30	−0.2963	0.9864	0.0038	0.0394	0.0011		
0.33	52:48	0	−0.1066	0.8014	0.0041	0.0282	0.0003	8.1669	12.7744
		10	−0.1861	0.8166	0.0005	0.0294	0.0011		
		20	−0.1910	0.9586	0.0013	0.0335	0.0006		
		30	−0.2386	0.9319	0.0038	0.0364	0.0013		
0.44	38:62	0	−0.1742	0.9536	0.0007	0.0274	0.00003	6.9418	15.3543
		10	−0.1739	0.9472	0.0055	0.0290	0.00008		
		20	−0.1499	0.9396	0.0013	0.0317	0.00005		
		30	−0.2963	0.9324	0.0038	0.0361	0.00003		

**Table 2:** The calculated free volume data for simulant lactose/water cells with 100 molecules at 0, 20, and 100 ps under various mimic *a_w_* (0.11 to 0.44) and temperatures.

Mimic Water Activities (*a_w_*)	Simulant Cell Size (*Ångstrom*)	Cell Density (g/cm^3^)	Temperature (*K*)	Time (ps)	Free Volume (*Ångstrom*^3^)	Percentage (%)
0.11	42.9 × 42.9 × 42.9	0.5	338	0	32,200.939	40.65
				20	42,319.684	53.42
				100	44,176.873	55.77
			348	0	32,067.512	40.48
				20	41,528.707	52.42
				100	44,364.481	56.00
			358	0	32,067.512	40.48
				20	40,812.000	51.52
				100	44,188.707	55.78
			368	0	32,067.512	40.48
				20	42,010.641	53.03
				100	44,653.483	56.37
0.22	40.7 × 40.7 × 40.7	0.5	313	0	27,575.943	40.93
				20	33,886.857	50.30
				100	36,417.298	54.06
			323	0	27,575.943	40.93
				20	35,110.157	52.12
				100	36,336.005	53.98
			333	0	27,575.943	40.93
				20	34,992.405	51.94
				100	37,323.439	55.40
			343	0	27,575.943	40.93
				20	35,689.584	52.98
				100	36,637.622	54.38
0.33	39.6 × 39.6 × 39.6	0.5	303	0	25,547.001	41.21
				20	31,309.400	50.51
				100	33,234.957	53.62
			313	0	25,547.001	41.21
				20	32,613.698	52.62
				100	33,937.488	54.75
			323	0	25,547.001	41.21
				20	31,759.772	51.24
				100	33,362.598	53.82
			333	0	25,547.001	41.21
				20	32,519.532	52.46
				100	33,265.822	53.67
0.44	36.0 × 36.0 × 36.0	0.5	286	0	18,752.591	39.98
				20	24,228.779	51.65
				100	24,856.389	52.99
			296	0	18,752.591	39.98
				20	23,792.236	50.72
				100	25,020.057	53.34
			306	0	18,752.591	39.98
				20	24,456.596	52.14
				100	25,199.910	53.72
			316	0	18,752.591	39.98
				20	24,338.806	51.89
				100	25,474.984	54.31

## Data Availability

The data presented in this study are available on request from the corresponding author. The data are not publicly available due to privacy restrictions.

## Supplementary Materials

The following supporting information can be downloaded at: https://www.mdpi.com/article/10.3390/foods14060928/s1, Figure S1: The changes and differences of *RMSD* in simulant lactose/water cells (100 molecules at mimic *a_w_* 0.11 and *T* − *T_g_* = 10 k) up to 1000 ps; Table S1: The calculated free volume data for simulant lactose/water cells with 100 molecules from 0 to 100 ps under various mimic *a_w_* (0.11 to 0.44) and temperatures.
