# Supplementary material for: Quantitative Analysis of Molecular Mobility in Amorphous Lactose Above Tg: A Novel Insight from Molecular Dynamic Simulation to Strength Parameter
Source: Foods. 2025 Mar 8;14(6):928. doi: 10.3390/foods14060928 (PMC11941022; doi:10.3390/foods14060928)
Supplement: Supplementary file 1 [file foods-14-00928-s001.zip › foods-3484934-supplementary.pdf]

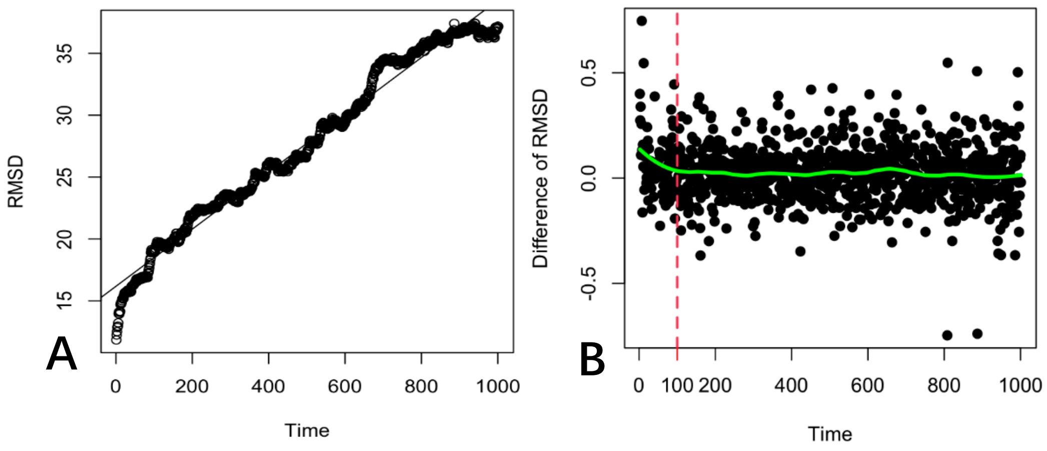

Figure S1. The changes (A) and differences (B) of *RMSD* in simulant lactose/water cells (100 molecules at mimic a_w 0.11 and $T\text{-}T_g$= 10k) up to 1000 ps.

Table S1. The calculated free volume data for simulant lactose/water cells with 100 molecules from 0 to 100 *ps* under various mimic a_w (0.11 to 0.44) and temperatures.

Water Activities (a_w)	Molecular Ratios (Lactose/Water)	Temperature (K)	Time (ps)	Free volume (Angstrom^3)	Percentage (%)
0.11	68:32	338k	0	32200.939	40.65
			20	42319.684	53.42
			40	43945.886	55.48
			60	43847.836	55.35
			80	44161.901	55.75
			100	44176.873	55.77
		343k	0	32067.512	40.48
			20	40690.392	51.37
			40	41711.563	52.66
			60	42133.913	53.19
			80	42424.626	53.56
			100	42512.679	53.67
		348k	0	32067.512	40.48
			20	41528.707	52.42
			40	43776.512	55.26
			60	44143.936	55.73
			80	44449.889	56.11
			100	44364.481	56.00
		353k	0	32067.512	40.48
			20	41318.997	52.16
			40	42723.704	53.93
			60	42482.800	53.63
			80	43229.021	54.57
			100	43200.711	54.53
		358k	0	32067.512	40.48
			20	40812.000	51.52
			40	43271.290	54.62
			60	43329.242	54.70
			80	43623.773	55.07
			100	44188.707	55.78
		363k	0	32067.512	40.48
			20	42602.792	53.78
			40	42601.050	53.78
			60	43137.546	54.46
			80	43714.282	55.18
			100	43810.099	55.30
		368k	0	32067.512	40.48

			20	42010.641	53.03
			40	43365.760	54.74
			60	44414.465	56.07
			80	44646.433	56.36
			100	44653.483	56.37
0.22	57:43	313k	0	27575.943	40.93
			20	33886.857	50.30
			40	35623.696	52.88
			60	36203.947	53.74
			80	36346.324	53.94
			100	36417.298	54.06
		318k	0	27575.943	40.93
			20	35675.228	52.95
			40	36609.891	54.34
			60	36624.597	54.36
			80	37154.124	55.15
			100	37026.643	54.96
		323k	0	27575.943	40.93
			20	35110.157	52.12
			40	36123.814	53.62
			60	36375.576	53.99
			80	36312.651	53.90
			100	36336.005	53.98
		328k	0	27575.943	40.93
			20	34436.209	51.12
			40	35050.560	52.03
			60	35121.123	52.13
			80	35849.407	53.21
			100	36163.904	53.68
		333k	0	27575.943	40.93
			20	34992.405	51.94
			40	36607.752	54.34
			60	36865.774	54.72
			80	36861.210	54.71
			100	37323.439	55.40
		338k	0	27575.943	40.93
			20	35883.461	53.26
			40	36300.133	53.88
			60	36410.786	54.05
			80	36589.038	54.31
			100	36591.542	54.31
		343k	0	27575.943	40.93
			20	35689.584	52.98
			40	36260.153	53.82

			60	36725.948	54.51
			80	36708.565	54.49
			100	36637.622	54.38
0.33	52:48	303k	0	25547.001	41.21
			20	31309.400	50.51
			40	32544.007	52.50
			60	32877.725	53.04
			80	33132.575	53.45
			100	33234.957	53.62
		308k	0	25547.001	41.21
			20	32075.247	51.75
			40	32875.755	53.04
			60	33057.438	53.33
			80	33345.236	53.80
			100	33589.514	54.19
		313k	0	25547.001	41.21
			20	32613.698	52.62
			40	33510.966	54.06
			60	33607.165	54.22
			80	33803.168	54.53
			100	33937.488	54.75
		318k	0	25547.001	41.21
			20	31712.665	51.16
			40	33233.580	53.62
			60	33278.924	53.69
			80	33466.742	53.99
			100	33354	53.81
		323k	0	25547.001	41.21
			20	31759.772	51.24
			40	33041.261	53.31
			60	33212.597	53.58
			80	33354.846	53.81
			100	33362.598	53.82
		328k	0	25547.001	41.21
			20	31583.471	50.95
			40	32723.784	52.79
			60	33176.670	53.52
			80	33222.576	53.60
			100	33405.717	53.89
		333k	0	25547.001	41.21
			20	32519.532	52.46
			40	33041.117	53.31
			60	33010.300	53.26
			80	33507.314	54.06

			100	33265.822	53.67
0.44	38:62	286 k	0	18752.591	39.98
			20	24228.779	51.65
			40	24715.644	52.69
			60	25031.847	53.36
			80	25138.713	53.59
			100	24856.389	52.99
		291	0	18752.591	39.98
			20	24263.621	51.73
			40	24474.417	52.18
			60	24376.214	51.97
			80	24837.428	52.95
			100	25071.148	53.45
		296	0	18752.591	39.98
			20	23792.236	50.72
			40	24395.399	52.01
			60	24678.300	52.61
			80	24904.801	53.09
			100	25020.057	53.34
		301	0	18752.591	39.98
			20	24284.105	51.77
			40	25022.415	53.34
			60	25260.337	53.85
			80	25704.869	54.80
			100	25554.724	54.48
		306	0	18752.591	39.98
			20	24456.596	52.14
			40	25128.928	53.57
			60	25098.129	53.51
			80	24943.412	53.18
			100	25199.910	53.72
		311	0	18752.591	39.98
			20	23873.709	50.90
			40	24432.277	52.09
			60	24633.096	52.51
			80	24816.944	52.91
			100	25112.534	53.54
		316	0	18752.591	39.98
			20	24338.806	51.89
			40	24546.490	52.33
			60	25279.137	53.89
			80	25579.540	54.53
			100	25474.984	54.31
